# Long‐acting injectable cabotegravir: implementation science needed to advance this additional HIV prevention choice

**DOI:** 10.1002/jia2.25963

**Published:** 2022-07-28

**Authors:** Heather‐Marie Ann Schmidt, Michelle Rodolph, Robin Schaefer, Rachel Baggaley, Meg Doherty

**Affiliations:** ^1^ Global HIV, Hepatitis and STIs Programme World Health Organization (WHO) Geneva Switzerland; ^2^ UNAIDS Regional Office for Asia and the Pacific Bangkok Thailand

1

A decade has passed since the World Health Organization (WHO) first recommended daily oral pre‐exposure prophylaxis (PrEP) as an additional HIV prevention option [1]. The global potential for PrEP to reduce new HIV infections has yet to be realized. An estimated 1.5 million new HIV infections occurred in 2020, a decline of only 31% since 2010 and significantly below the 75% reduction target [2]. Scale‐up of oral PrEP has begun to accelerate [3], yet only 28% of the 3 million PrEP users target for 2020 was reached [2]. Important advances in PrEP delivery are now beginning to expand access and uptake, including differentiated service delivery [4] and greater choices, including event‐driven oral PrEP, and the first long‐acting PrEP product, the dapivirine vaginal ring [5]. However, to further expand HIV prevention coverage and reduce new infections, rapid scale‐up of existing PrEP options and the introduction of additional PrEP products, alongside other HIV prevention interventions, are urgently needed.

Long‐acting injectable cabotegravir (CAB‐LA) is a new, safe and highly efficacious additional PrEP choice. Results from two randomized controlled trials (HPTN 083 and HPTN 084) suggested an estimated 66% relative reduction in HIV risk among cisgender men and transgender women who have sex with men [6] and 88% reduction among cisgender women compared with oral PrEP [7]. Long‐acting injectable products have been found to be acceptable and often preferred in studies examining community PrEP preferences [8, 9, 10, 11, 12]. As such, CAB‐LA may reach new clients for HIV prevention and be a more acceptable choice for people who have concerns about oral PrEP or other prevention options or difficulty taking these as prescribed.

In July 2022, WHO is releasing a recommendation about the use of CAB‐LA for PrEP to encourage availability in low‐ and middle‐income countries (LMICs) and support inclusion in national guidelines and PrEP programmes [13]. This WHO recommendation will let manufacturers apply for inclusion on the WHO list of prequalified medicinal products, allowing agencies, such as the Global Fund, UNICEF and Unitaid, to procure CAB‐LA. However, important issues regarding use in LMICs remain unresolved, and a bold programme of implementation research is urgently needed for CAB‐LA to be implemented acceptably, safely and effectively at scale to achieve significant impact.

Current experience with CAB‐LA provision is largely limited to clinical trial settings. Evidence is lacking about effective models for providing CAB‐LA in real‐world settings, especially for populations either not included or under‐represented in the trials, including sex workers, people who inject drugs and transgender men, and others living in diverse settings and geographies.

There is also a need for further studies to support obtaining additional data about CAB‐LA safety in some populations. While pharmacokinetic analyses did not show any clinically relevant effect by gender [14], research needs to ensure that there is no interaction between CAB‐LA and gender‐affirming hormone use among trans and gender diverse populations. Alternative injection sites should be considered for those unable to receive intramuscular gluteal injections, such as those with buttock implants. In addition, the acceptability, feasibility, safety and training needs for individuals to self‐administer injections could be explored. This approach has been acceptable and feasible in other contexts, including for hormone injections, and may increase convenience and acceptability of CAB‐LA and reduce service visits. Furthermore, while the limited evidence available suggests that CAB‐LA is safe during pregnancy and breastfeeding, further large‐scale implementation and post‐market surveillance will be needed to understand whether there are any serious adverse events associated with CAB‐LA use during pregnancy and breastfeeding.

Where to deliver CAB‐LA for maximum impact and acceptability must also be explored, including sexual and reproductive health services, ante‐ and post‐natal services, and community settings. Differentiated service delivery models have been crucial to oral PrEP access globally by providing person‐centred services, adapted to the needs and preferences of individuals and communities. CAB‐LA must be offered as a choice alongside a range of HIV prevention options, including oral PrEP, and must not create additional barriers to service access or reduce integration in settings where task‐sharing with non‐physician providers is important, such as community‐led and other differentiated services providing oral PrEP.

In addition, optimal HIV testing strategies for CAB‐LA need to be established to support CAB‐LA integration with existing services. HIV testing before the initiation of CAB‐LA and during use is important to minimize the likelihood of a person living with HIV starting CAB‐LA, to detect seroconversions early, and to reduce the risk of drug resistance that could compromise the effectiveness of integrase strand transfer inhibitor (INSTI) drugs used widely for HIV treatment, notably dolutegravir. Although only a small number of people acquired HIV while taking CAB‐LA in the trials [6, 7], preliminary findings of mathematical modelling suggest that widespread CAB‐LA use could considerably increase the prevalence of INSTI resistance [15]. However, the modelling also suggests that population‐level HIV‐related mortality would likely be lower due to decreases in HIV incidence and prevalence [15]. The United States Food and Drug Administration, the first regulatory authority to approve CAB‐LA, recommends nucleic acid amplification testing (NAAT) prior to initiation and follow‐up. NAAT could detect HIV infection earlier than third‐generation antibody tests typically used in LMIC national testing algorithms. However, as confirmatory HIV testing is required for diagnosis, earlier detection with NAAT may not result in an earlier diagnosis. Moreover, NAATs are not readily available in many LMICs, as most do not have regulatory approval for HIV diagnosis.Comparatively long turnaround times and higher costs limit the feasibility of this approach. Furthermore, while earlier HIV detection could identify seroconversion before drug resistance developed, the extent to which using NAAT testing would prevent the development of INSTI resistance and cross‐resistance to dolutegravir is still unknown. The optimal testing algorithm for real‐world implementation of CAB‐LA is, therefore, uncertain. The risk of delayed identification of HIV infection and potential increase in HIV drug resistance (theoretically prevented with NAAT) must be balanced with the benefits of wider CAB‐LA access and decreasing new HIV infections (using current national testing algorithms).

As a new HIV prevention option, communities and providers in many settings currently have limited awareness about CAB‐LA. Programmes must invest in awareness and demand‐raising activities to support introduction using positive, gain‐framing messaging to position CAB‐LA alongside other HIV prevention choices. While potential PrEP users are interested in injectable PrEP [8, 9, 10, 11, 12], it is unclear how choices will translate into the uptake of CAB‐LA and how people from different populations and geographies may choose to start, stop and restart CAB‐LA, and switch between PrEP options. Indeed, while people supported to continue CAB‐LA had high adherence to bi‐monthly injections at 91.5% and 93% for HPTN 083 and 084, respectively [6, 7], how effective use can be supported outside trial settings remains to be determined.

Inclusion within national programmes will also depend on demonstrated cost‐effectiveness. CAB‐LA must be offered at a low cost to enable LMIC implementation, but the costs of other services within the basic PrEP package are also important, including regular testing for HIV and other sexually transmitted infections.

The WHO recommendation on offering CAB‐LA as HIV prevention for people at substantial risk for HIV will provide an additional choice for HIV prevention. CAB‐LA also provides us with a unique opportunity to leverage the excitement for this new option to strengthen and expand HIV prevention programmes. But to achieve the full potential of CAB‐LA, all sectors––communities, ministries of health, clinical providers, researchers, implementers, donors and manufacturers––must work together to conduct meaningful implementation science as part of PrEP scale‐up programmes and ensure that provision of CAB‐LA enhances the availability, accessibility, acceptability and affordability of effective HIV prevention services.

## COMPETING INTERESTS

The authors declare no competing interests.

## AUTHORS’ CONTRIBUTIONS

HMAS, RS, MR and RB contributed to the writing of the manuscript and provided key conceptual elements. All authors have read, reviewed, contributed to and approved the final manuscript for this Viewpoint.

## FUNDING

No funding was provided for this article.

## DISCLAIMER

The authors alone are responsible for the views expressed in this article and they do not necessarily represent the views, decisions or policies of the WHO or UNAIDS.
